# Comparative Plastid Genomes of *Primula* Species: Sequence Divergence and Phylogenetic Relationships

**DOI:** 10.3390/ijms19041050

**Published:** 2018-04-01

**Authors:** Ting Ren, Yanci Yang, Tao Zhou, Zhan-Lin Liu

**Affiliations:** 1 Key Laboratory of Resource Biology and Biotechnology in Western China (Ministry of Education), College of Life Sciences, Northwest University, Xi’an 710069, China; renting92@stumail.nwu.edu.cn (T.R.); yycjyl1@gmail.com (Y.Y.); 2 School of Pharmacy, Xi’an Jiaotong University, Xi’an 710061, China; zhoutao196@mail.xjtu.edu.cn

**Keywords:** plastid genome, phylogenetic relationship, *Primula*, repeat, sequence divergence

## Abstract

Compared to traditional DNA markers, genome-scale datasets can provide mass information to effectively address historically difficult phylogenies. *Primula* is the largest genus in the family Primulaceae, with members distributed mainly throughout temperate and arctic areas of the Northern Hemisphere. The phylogenetic relationships among *Primula* taxa still maintain unresolved, mainly due to intra- and interspecific morphological variation, which was caused by frequent hybridization and introgression. In this study, we sequenced and assembled four complete plastid genomes (*Primula handeliana*, *Primula woodwardii*, *Primula knuthiana*, and *Androsace laxa*) by Illumina paired-end sequencing. A total of 10 *Primula* species (including 7 published plastid genomes) were analyzed to investigate the plastid genome sequence divergence and their inferences for the phylogeny of *Primula*. The 10 *Primula* plastid genomes were similar in terms of their gene content and order, GC content, and codon usage, but slightly different in the number of the repeat. Moderate sequence divergence was observed among *Primula* plastid genomes. Phylogenetic analysis strongly supported that *Primula* was monophyletic and more closely related to *Androsace* in the Primulaceae family. The phylogenetic relationships among the 10 *Primula* species showed that the placement of *P. knuthiana–P. veris* clade was uncertain in the phylogenetic tree. This study indicated that plastid genome data were highly effective to investigate the phylogeny.

## 1. Introduction

*Primula* is the largest genus in the family Primulaceae with approximately 500 species [1,2], where they are especially rich in the temperate and arctic areas of the Northern Hemisphere, with only a few outliers found in the Southern Hemisphere. China is the center of *Primula* diversity and speciation with over 300 species [1,3]. Many *Primula* species are grown widely as ornamental and landscape plants because of their attractive flowers and long flowering period. Therefore, *Primula* is reputed to be one of the great garden plant genera throughout the world [2,3].

As a typical cross-pollinated plant with heterostyly, *Primula* has been a particular focus of many botanists, and various studies are involved in hybridization [4], pollination biology [5,6], and distyly [7,8]. According to morphological traits, the taxonomic study of *Primula* has been revised for several times. Smith and Fletcher (1947) firstly proposed an infrageneric system with a total of 31 sections [9]. Considering some putative reticulate evolutionary relationships, Wendelbo (1961) posed a revised system with seven subgenera [10]. Richards (1993) later amended Wendelbo’s version and classified six subgenera [11]. Hu and Kelso (1996) delimited the Chinese *Primula* species into 24 sections [1]. Recently, numerous molecular phylogenetic works of the genus *Primula* have also been conducted by using plastid and/or nuclear gene fragments [12,13,14]. These studies have greatly advanced our understanding of the evolutionary history of *Primula* species. However, the phylogenetic relationships within the genus *Primula* are still uncertain, mainly due to intra- and interspecific morphological variation, which was caused by frequent hybridization and introgression [1,2,14]. Further research has been hindered by the insufficient information of the traditional DNA markers, such as one or few chloroplast gene fragments, and by the complex evolutionary relationships in *Primula*. Therefore, more sequence resources and genome data are required in order to obtain a better understanding of the phylogeny of the genus *Primula*.

In general, the plastid genome in angiosperms is a typical quadripartite structure, where the size ranges from 115 to 165 kb, with two copies of inverted repeat (IR) regions separated by a large single copy (LSC) region and a small single copy (SSC) region [15]. Approximately 110–130 distinct genes are located along the plastid genome [16]. Most of these are protein-coding genes, the remainder being transfer RNA (*tRNA*) or ribosomal RNA (*rRNA*) genes [16]. Due to its particular advantages—such as small size, uniparental inheritance, low substitution rates, and high conservation in terms of the gene content and genome structure [17,18]—the plastid genome is considered a very promising tool for phylogenetic studies [19,20]. Significant advances in next-generation sequencing technology made it fairly inexpensive and convenient to obtain plastid genome sequences [21,22]. As a result, phylogenomic analyses have also been greatly facilitated. For example, the plastid phylogenomic analyses supported Tofieldiaceae as the most basal lineage within Alismatales [23]. The relationships between wild and domestic *Citrus* species could also be resolved with 34 plastid genomes [24]. Similarly, 142 plastid genomes were used to successfully infer deep phylogenetic relationships and the diversification history of Rosaceae [25]. These studies strongly indicate that plastid phylogenomics is helpful in determining the phylogenetic positions of various questionable lineages of angiosperms.

In the present study, we analyzed the complete plastid genomes of 10 *Primula* species including 7 published plastid genomes and 3 new data (*Primula handeliana*, *Primula woodwardii*, and *Primula knuthiana*) by using Illumina sequencing technology. Our primary aims were to: (1) compare the complete plastid genomes of 10 *Primula* species; (2) document that the extent of sequence divergence among the *Primula* plastid genomes; and (3) increase more sequence resources and genome information for investigating the phylogeny in genus *Primula*. The complete plastid genome of *Androsace laxa* from a closely related genus was used as the outgroup in the phylogenomic analysis of genus *Primula*. This study will not only contribute to further studies on the phylogeny, taxonomy, and evolutionary history of the genus *Primula*, but also provide insight into the plastid genome evolution of *Primula*.

## 2. Results

### 2.1. Genome Features

The sizes of the plastid genomes of the 10 *Primula* species ranged from 150,856 bp to 153,757 bp, where they had a typical quadripartite structure, including a LSC region (82,048–84,479 bp) and a SSC region (17,568–17,896 bp) separated by a pair of IR regions (25,182–25,855 bp) (Table 1). In the 10 *Primula* plastid genomes, gene content was similar and gene order was identical. The *Primula* plastid genomes contained about 130–132 genes, including 85–86 protein-coding genes, 37 *tRNA* genes, and 8 *rRNA* genes (Tables 1 and S4). The *accD* gene was a pseudogene in *P. sinensis*, whereas it was missing in *P. persimilis* and *P. kwangtungensis*. The *P. poissonii* plastid genome contained a pseudogene (*infA*). Among these genes, 15 genes harbored a single intron (*trnA-UGC*, *trnG-UCC*, *trnI-GAU*, *trnK-UUU*, *trnL-UAA*, *trnV-UAC*, *atpF*, *ndhA*, *ndhB*, *petB*, *petD*, *rpoC1*, *rpl2*, *rpl16*, and *rps16*) and three genes (*pafI*, *clpP*, and *rps12*) harbored two introns. Seven *tRNA* genes, seven protein-coding genes, and all four *rRNA* genes were completely duplicated in the IR regions (Table 1). *trnk-UUU* had the largest intron (2487–2568 bp) containing the *matK* gene. The GC contents of the LSC, SSC, and IR regions, as well as those of the whole plastid genomes, were nearly identical in the 10 *Primula* plastid genomes (Table 1). The complete plastid genome of *A. laxa* was 151,942 bp in length and contained 132 genes (Table 1). The overall GC content of the *A. laxa* plastid genome was 37.3%, and the corresponding values for the LSC, SSC, and IR regions were 35.2, 30.9, and 42.7%, respectively (Table 1).

### 2.2. Codon Usage Analysis

Codon usage plays a crucial role in evolution of plastid genome. Here, we first analyzed codons of the protein-coding genes in the 10 *Primula* plastid genomes. The number of encoded codons ranged from 25,781 (*P. sinensis*) to 26,505 (*P. knuthiana*) (Table S5). Detailed codon analysis showed that the 10 *Primula* species had similar codon usage and relative synonymous codon usage (RSCU) values (Table S5). Leucine and Cysteine were the highest (2743–2823 codons) and lowest (280–298 codons) frequent amino acids in these species, respectively (Table S5). RSCU > 1 denotes that the codon is biased and used more frequently, RSCU = 1 shows that the codon has no bias, and RSCU < 1 indicates that the codon is used less frequently. All 10 *Primula* plastid genomes had 30 biased codons with RSCU > 1 (Table S5). The biased codons had higher representation rates for A or T at the third codon position in a similar manner to the majority of angiosperm plastid genomes. Except for TTG, all of the types of biased codons (RSCU > 1) ended with A or T. The GC% was quite different at the three codon positions (Table S6). The average values of GC% for the first, second, and third codon positions of 10 *Primula* species were 45.3, 37.9, and 29.2%, respectively (Table S6). The observation of GC% level also indicated that plastid genome in *Primula* was a strong bias toward A or T at the third codon position.

### 2.3. Analysis of Repeat Elements

**Figure 1:**
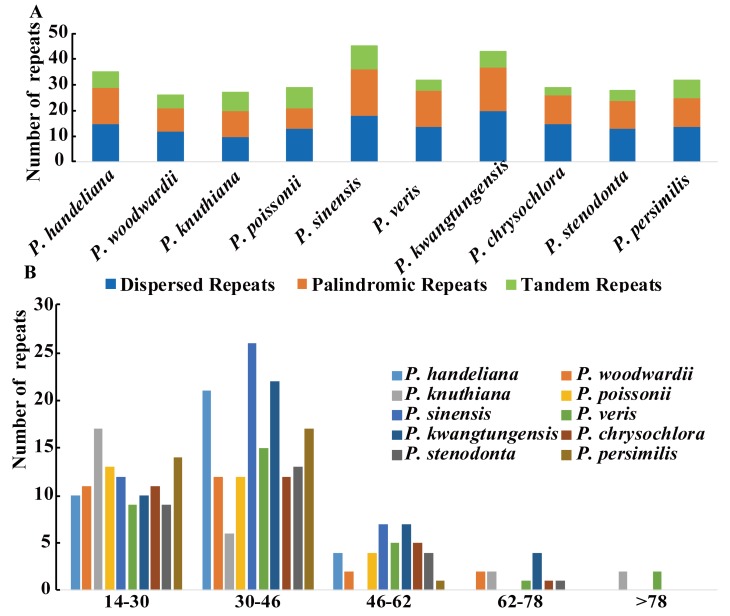
The type of repeated sequences in the 10 *Primula* plastid genomes. (**A**) Number of three repeat types; (**B**) number of repeat sequences by length.

Three categories of repeats (dispersed, palindromic, and tandem repeats) were identified in the 10 *Primula* plastid genomes. We detected 326 repeats in total comprising 144 dispersed, 123 palindromic, and 59 tandem repeats (Figure 1A and Table S7). Among them, repeats of *P. sinensis* (45) were the greatest and that of *P. woodwardii* (26) were the lowest (Figure 1A and Table S7). The majority of the repeats (95.4%) ranged in size from 14 to 62 bp (Figure 1B and Table S7). Repeats located in intergenic spacer (IGS) and intron regions comprised 44.2% (144 repeats) of the total repeats and 47.8% (156 repeats) were located in *ycf2* gene, whereas only a minority were located in other coding DNA sequence (CDS) regions, such as *psaB*, *trnS-GCU*, *ycf1*, *rpoB*, *ndhF,* etc. (Table S7).

**Figure 2:**
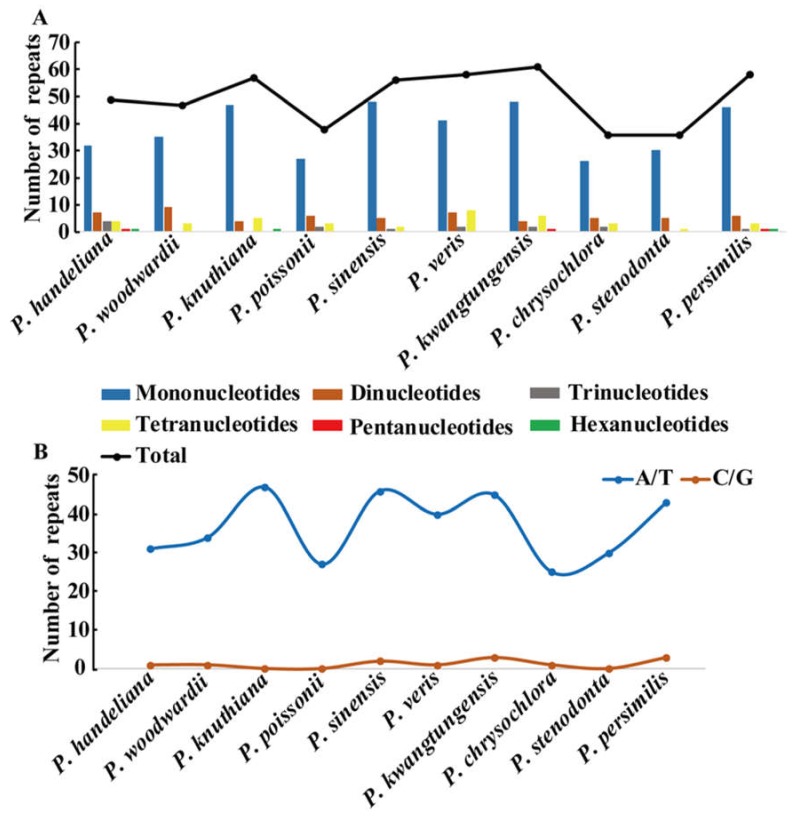
Simple sequence repeats (SSRs) in the 10 *Primula* plastid genomes. (**A**) Number of SSR types; (**B**) number of mononucleotide A/T and G/C SSRs.

A total of 496 simple sequence repeats (SSRs) measuring at least 10 bp in length were also analyzed (Figure 2A and Table S8). Among these SSRs, the mononucleotide, dinucleotide, trinucleotide, tetranucleotide, pentanucleotide, and hexanucleotide SSRs were all detected. The mononucleotide SSR were the richest with a proportion of 76.6%, followed by dinucleotide SSR (11.7%), tetranucleotide SSR (7.7%), and trinucleotide SSR (2.8%) (Figure 2A and Table S8). We only detected six pentanucleotide and hexanucleotide SSRs in the 10 *Primula* cp genomes (Figure 2A and Table S8). Unsurprisingly, the mononucleotide A/T SSR occupied the highest portion (368; 74.2%) (Table S8). The number of mononucleotide A/T SSR was significantly higher than that of the mononucleotide G/C SSR (Figure 2B and Table S8). Furthermore, most of the SSRs were found in IGS regions (56.9%), followed by CDS regions (25%), and intron regions (17.9%) (Table S8). SSRs located in the CDS region were mainly found in the *ycf1* gene.

### 2.4. IR/SC Boundary and Genome Rearrangement

**Figure 3:**
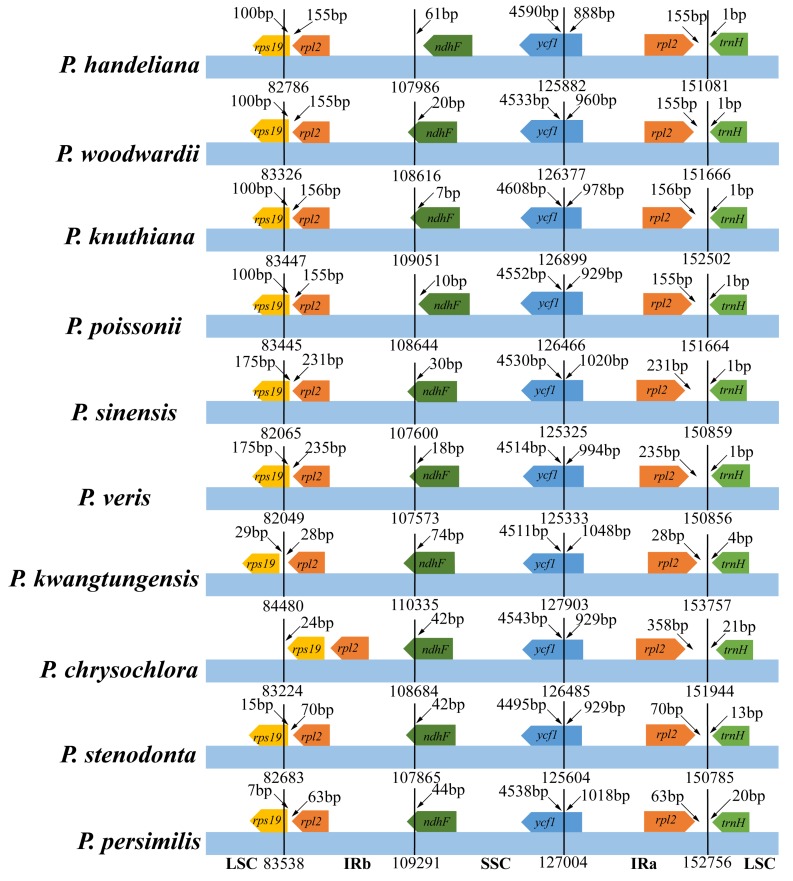
Comparison of the LSC, IR, and SSC border regions among the 10 *Primula* plastid genomes. Number above the gene features means the distance between the ends of genes and the borders sites. These features are not to scale.

The IR/SC boundary contents of 10 *Primula* plastid genomes were compared (Figure 3). The gene content and gene order were conserved at the IR/SC boundary, but the *Primula* plastid genomes exhibited more obvious differences. In the *P. kwangtungensis* plastid genome, the *rps19* gene was located entirely in the LSC, whereas IRb extended in a variable manner 7–175 bp into the *rps19* gene in all the other species. In the *P. chrysochlora* plastid genome, IRb even crossed completely into the *rps19* gene. IRb extended 7–74 bp in a variable manner into the *ndhF* genes, except in the *P. handeliana* and *P. poissonii* plastid genomes. In all of the *Primula* plastid genomes, IRa extended into the *ycf1* genes, where the smallest and largest extensions occurred in the *P. handeliana* (888 bp) and *P. kwangtungensis* (1048 bp) plastid genomes. The whole-genome alignment of the 10 *Primula* plastid genomes showed no rearrangement events in *Primula* (Figure S1).

### 2.5. Sequence Divergence

**Figure 4:**
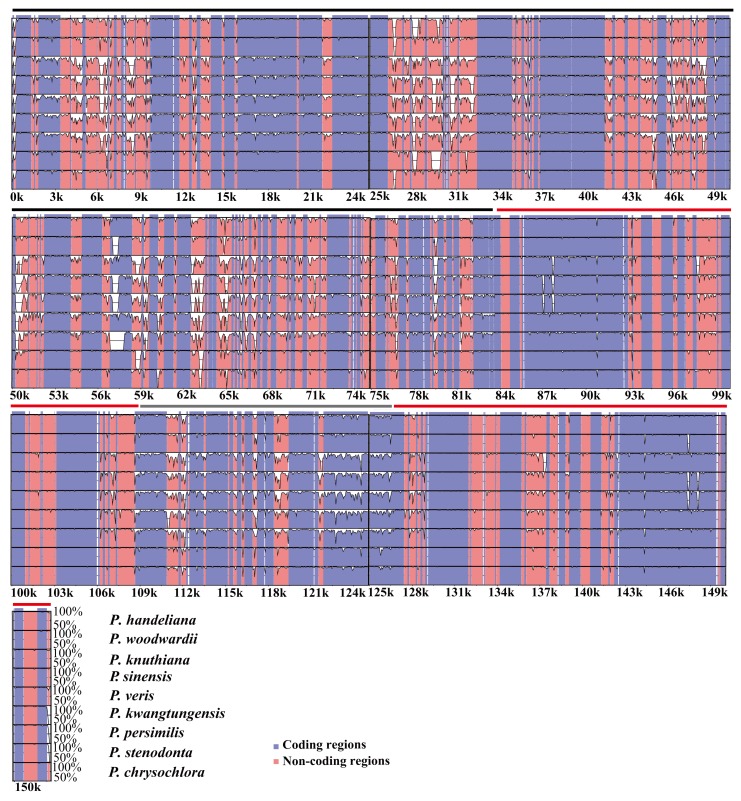
Sequence identity plot of the 10 *Primula* plastid genomes, with *Primula* poissonii as a reference. The *y*-axis represents % identity ranging from 50% to 100%. Coding and non-coding regions are marked in purple and pink, respectively. The red, black, and gray lines show the IRs, LSC, and SSC regions, respectively.

To investigate the levels of sequence divergence, the 10 *Primula* plastid genomes were plotted using mVISTA with *P. poissonii* as the reference (Figure 4). The *Primula* plastid genomes exhibited moderate sequence divergence (Figure 4). As expected, coding and IR regions exhibited more sequence conservation than non-coding and SC regions, respectively (Figure 4). We then calculated the percentage of variable characters for each coding region and non-coding regions with an alignment length of more than 200 bp (Table S9). The average percentage of variation in non-coding regions is 0.38, which was significantly higher than that in the coding regions (0.088 on average; Table S9). The *accD* gene contained various indels and it was a pseudogene in *P. sinensis* and missing in *P. persimilis* and *P. kwangtungensis*, which may have caused the most divergent coding region. In addition, 15 genes had a percentage of variation greater than 0.10 (Table S9), i.e., *ycf1* (0.23), *matK* (0.18), *ycf15* (0.17), *ndhF* (0.17), *rpl33* (0.16), *rpl22* (0.16), *rps16* (0.15), *rps8* (0.12), *ccsA* (0.12), *rps15* (0.12), *rpoC2* (0.11), *psbH* (0.11), *ndhD* (0.11), *rpoA* (0.10), and *ndhA* (0.10). Among the 16 genes with higher percentages of variation, 15 genes were found in SC regions and only one gene in IR regions (Table S9). The average percentages of variations in the LSC, SSC, and IR regions were 0.42, 0.43 and 0.15 in the non-coding regions, while the corresponding values in the coding regions were 0.09, 0.11, and 0.04, respectively (Table S9). All of the results demonstrated that the IR regions were more conserved than the SC regions. The overall sequence divergence based on the p-distance among the 10 *Primula* species was 0.028143 (Table S10). The pairwise p-distance between the 10 species ranged from 0.005857 to 0.041629 (Table S10). These results suggested that moderate sequence divergence has occurred within the genus *Primula*.

### 2.6. Phylogenomic Analysis

**Figure 5:**
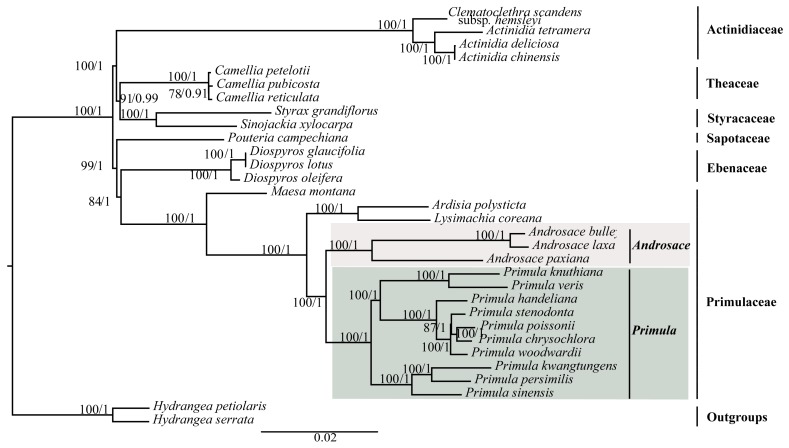
Phylogenetic relationship of the 31 species inferred from ML and BI analyses based on 76 shared protein-coding genes. The numbers near each node are bootstrap support values and posterior probability. *Hydrangea petiolaris* and *Hydrangea serrata* were used as the outgroups.

To investigate the phylogenetic position of *Primula*, three datasets (76 shared protein-coding genes, codon positions 1 + 2, and codon position 3) were used to conduct the BI and ML analyses (Figure 5 and Figure S2). The selected models of each dataset were shown in Table 2. Support values were generally high for almost all relationships inferred from 76 shared protein-coding genes (the support values had a range of 78/0.91–100/1) (Figure 5). All phylogenetic trees clearly identified that *Primula* was monophyletic and more closely related to *Androsace* with high support values (Figure 5 and Figure S2).

**Figure 6:**
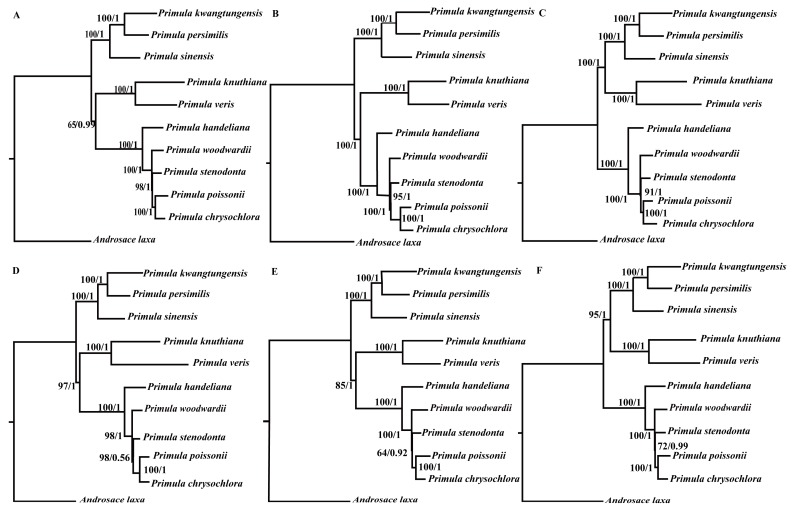
Phylogenetic relationships of the 10 *Primula* species and *A. laxa* inferred from ML and BI analyses. (**A**) Whole plastid genomes; (**B**) protein-coding regions; (**C**) introns and intergenic spacer regions; (**D**) IR regions; (**E**) SSC regions; and (**F**) LSC regions. The numbers near each node are bootstrap support values and posterior probability.

We then constructed six datasets (whole plastid genome, protein-coding regions, LSC, SSC, IRs, and introns & intergenic spacers) to analyze the phylogenetic relationships among the members of the genus *Primula*. The plastid genome of *A. laxa* was used as the outgroup. The selected models for each dataset used in BI and ML analyses were displayed in Table 2. The different datasets generally produced congruent phylogenetic trees (two topological structures) with moderate to high support values (Figure 6). All of the phylogenetic trees showed that *P. stenodonta*, *P. poissonii*, and *P. chrysochlora* formed a monophyletic group, where they belong to Sect. *Proliferae*. Although *P. woodwardii* and *P. handeliana* belong to Sect. *Crystallophlomis*, they were not monophyletic. *P. kwangtungensis*, *P. persimilis*, and *P. sinensis* belong to different sections, but they clustered together in the phylogenetic trees. In addition, *P. knuthiana* was more closely related to *P. veris* than other *Primula* species, but their placements varied in topological structure.

## 3. Discussion

### 3.1. Evolution of the Plastid Genome

Most angiosperm plastid genomes are highly conserved in terms of their gene content and order, but gene loss (deletion or production of pseudogenes) has occurred in several angiosperm lineages [26,27]. In our study, the *accD* gene was found in seven *Primula* plastid genomes, while it was a pseudogene in *P. sinensis* and was missing in *P. persimilis* and *P. kwangtungensis*. The *accD* gene encodes the acetyl-CoA carboxylase subunit D, which has been lost either partially or completely from some members of the Poales and Acoraceae [28]. The *infA* gene was a pseudogene in *P. poissonii* plastid genome, but it has been entirely lost from the other *Primula* plastid genome. The *infA* gene encodes translation initiation factor 1, which assists with the assembly of the translation initiation complex [18]. Similar events have also occurred in other angiosperm plastid genomes, such as those of *Hagenia abyssinica* [29] and *Morella rubra* [30], although the plastid genome of *A. laxa* contains the *infA* gene. The photosystem assembly factors (*ycf3* and *ycf4*) that act on photosystem I complex [31,32] should be renamed as *pafI* and *pafII* (respectively) according to recent studies [18]. Here, we use the new names of the two genes in both *Primula* and *A. laxa* plastid genomes.

IRs are the most conserved regions in the plastid genomes, where the contraction and expansion of the IR regions have occurred frequently. Our results indicated more obvious differences at the IR/SC boundaries. Particularly, in the *P. kwangtungensis* plastid genome, the *rps19* gene was located entirely in the LSC. By contrast, IRb extended into the *rps19* gene and it even completely crossed the *rps19* gene in the *P. chrysochlora* plastid genome. In addition, IRa extended into the *ycf1* genes where the smallest and largest extensions occurred in *P. handeliana* (888 bp) and *P. kwangtungensis* (1048 bp). The expansions of IRs into the *rps19* gene and *ycf1* gene have been also observed in *Cardiocrinum* [33] and *Amana* [34]. IR regions contraction and expansion events are relatively common evolutionary phenomena in plants [35]. Moreover, IR region loss was observed in some species [36,37].

Large and complex repeat sequences may play important roles in the arrangement and recombination of the plastid genome [38,39]. In all, 326 repeats were detected in the 10 *Primula* plastid genomes. Compared with other angiosperm species [40], this number is relatively small. Most of repeats ranged in size from 14 to 62 bp and almost all were not large repeats (>100 bp), which were in a similar manner to those reported in other plants [41,42,43]. *Pelargonium*, *Trifolium*, and *Trachelium*, the most highly rearranged plastid genomes contain a high frequency of large repeats (>100 bp) [44]. Our study revealed that no rearrangement events occurred in *Primula*, we thus deduced that may be mainly ascribed to no large repeats in these 10 *Primula* plastid genomes. Repeats located in *ycf2* gene occupied 47.8% of the total repeats. The *ycf2* gene is the largest gene in the *Primula* plastid genomes with over 6000 bp in length, and is completely duplicated in the IR regions. This phenomenon has also been reported in *Cardiocrinum* [33]. SSRs are highly polymorphic, and thus they are employed as molecular markers for population genetics and phylogenetic investigations [45,46]. Notably, the majority of the SSRs in the 10 *Primula* plastid genomes were the mononucleotide A/T SSRs (74.2%), which supports previous reports that SSRs in the plastid genome generally comprise short polyadenine (polyA) or polythymine (polyT) repeats [47,48]. Most of the SSRs were found in IGS regions (56.9%), followed by CDS regions (25%) and introns (17.9%). The CDS region with the highest number of SSRs was *ycf1*, as found in other species, such as *Cardiocrinum* [33] and *Vigna radiata* [49]. In the 10 *Primula* plastid genomes, the *ycf1* gene usually spanned the small single copy (SSC) and the inverted repeat a (IRa) region. It is very interesting that all but two of the SSRs in the *ycf1* gene are distributed in the SSC region. It is possible because the section of *ycf1* gene in the IRa region is shorter (less than one kilobase long) than these in SSC region (more than four kilobase long) [50]. The cpSSRs reported here would be potential molecular markers for future studies of *Primula* species.

According to the results obtained using mVISTA, the *Primula* plastid genomes exhibited moderate sequence divergence, especially in the non-coding regions. Our study showed that the coding regions were more conserved than the non-coding regions, as found in many plants [41,42,43]. Besides, the IR regions were more conserved than the SC regions as previous studies [51]. This fact that the two IR regions were less variable was attributed to the conservation of the ribosomal RNA genes, which comprised about one-third of the IR region in the plastid genomes [17]. The p-distance results also confirmed that moderate sequence divergence exists within the genus *Primula*. Compared with related herbaceous plants, trees, and shrubs generally have relatively long generation times and low rates of molecular evolution [52]. Herbs have shorter generation times and show much higher rates of molecular change and variance in rates [52]. The genetic diversity of heterotypic flower plants is higher than that of self-pollinated plants, indicating that genetic variation is easy to occur in interspecific and intraspecific species of heterotypic flower plants [53]. Therefore, the moderate sequence divergence probably be related to biology characteristics of these *Primula* species, such as perennial herbs, shorter generation times, cross-pollination, distyly, etc.

### 3.2. Phylogenetic Relationships

Plastid genomes have been successfully used to resolve the phylogenetic relationships in plant groups [23,25,54]. In this study, we used two methods (ML and BI) to construct the phylogenetic trees. We used three datasets to investigate the phylogenetic position of *Primula*. All of the phylogenetic trees indicated that *Primula* was monophyletic and more closely related to *Androsace* in Primulaceae family. Besides, in the genus *Primula*, all of the phylogenetic trees showed that Sect. *Proliferae* (*P. chrysochlora*, *P. poissonii*, and *P. stenodonta*) formed a monophyletic group and *P. chrysochlora* was closely related to *P. poissonii* [55]. Both *P. woodwardii* and *P. handeliana* belong to Sect. *Crystallophlomis*, but they did not have the closest relationship. The phylogenetic trees indicated that *P. woodwardii* and Sect. *Proliferae* were sister groups, then they clustered with *P. handeliana* in the same clade. Section *Crystallophlomis* and *Proliferae* were clustered into one clade in this study, which was also supported by karyotype study [56], but was inconsistent with the morphological work [9]. The placement of *P. knuthiana*-*P. veris* clade was uncertain in the phylogenetic tree. This was partly due to the rapid evolution of genus *Primula* [14,57]. The lack of samples might also affect the results of the phylogenetic analysis. In fact, for this large genus, our study could not fully clarify the relationships among *Primula* species due to the limited taxa sampled. Hence, more species and comprehensive analyses should be included in the future phylogenetic studies of *Primula* species. All in all, our analysis based on plastid genomes provides a valuable resource that should facilitate future phylogeny, taxonomy, and evolutionary history studies of this genus.

## 4. Materials and Methods

### 4.1. Plant Materials and DNA Extraction

The four plant materials (*Primula handeliana*, *Primula woodwardii*, *Primula knuthiana*, and *Androsace laxa*) used in this study were sampled from Taibai Mountain (Shaanxi, China; 107.77 °E, 33.95 °N). Total genomic DNA was extracted from silica-dried leaves with a modified CTAB method [58] by Biomarker Technologies Inc., Beijing, China. Voucher specimens were deposited in the Key Laboratory of Resource Biology and Biotechnology, Northwest University. All of the newly generated complete plastid genome sequences were deposited in GenBank (https://www.ncbi.nlm.nih.gov) (Table 1). The complete plastid genomes of *Primula poissonii* (NC_024543) [59], *Primula sinensis* (NC_030609) [57], *Primula veris* (NC_031428) [60], *Primula kwangtungensis* (NC_034371) [61], *Primula chrysochlora* (KX668178) [55], *Primula stenodonta* (KX668176) [62], and *Primula persimilis* (KX641757) [63] were recovered in order to conduct follow-up analysis (Table S1).

### 4.2. Illumina Sequencing, Assembly, and Annotation

Whole-genome sequencing was performed using the 150 bp pair-end sequencing method with the Illumina Hiseq 2500 Platform by Biomarker Technologies Inc. (Beijing, China). First, the raw Illumina reads were quality trimmed using the NGSQC Toolkit_v2.3.3 [64] with the default cutoff values. The clean reads were then subjected to reference-guided assembly with the MIRA v4.0.2 program [65] (parameters: job = genome, mapping, accurate; technology = solexa; segment_placement = FR). We used *Primula poissonii* (NC_024543) and *Androsace bulleyana* (KU513438) as reference genomes to assemble the *Primula* species and *A. laxa*, respectively. The resultant contigs were further assembled using a baiting and iteration method based on MITObim v1.8 [66] with default parameters. In addition, we also used the SPAdes v3.6.2 [67] (*k* = 33, 55, 77) to assemble the resultant clean reads of four species. We performed de novo assembly in order to verify the validity and accuracy of assembly results. Finally, a few gaps containing some ambiguous bases “N” and low-coverage regions in the assembled plastid genomes were confirmed by PCR-based Sanger sequencing. The primer pairs were designed online with the Primer3 program [68] and listed in the Supplementary Table S2. All of the genes were annotated using Dual Organellar Genome Annotator (DOGMA) software [69] with the default parameters. We then corrected the annotations with the GENEIOUS R8.0.2 program (Biomatters Ltd., Auckland, New Zealand) based on comparisons with related species. Codon usage and relative synonymous codon usage (RSCU) [70] value were estimated for all exons in the protein-coding genes with the CodonW v1.4.2 program [71].

### 4.3. Identification of Repeat Sequences

We used the online REPuter program [72] to identify dispersed and palindromic repeats with a minimum repeat size of 30 bp and two repeats comprising not less than 90% (Hamming distance = 3). Tandem repeats were detected using the Tandem Repeat Finder program [73] by setting two, seven, and seven as the alignment parameters for match, mismatch, and indels, respectively. The minimum alignment score and maximum period size were 80 and 500, respectively. Simple sequence repeats (SSRs) were detected using the Perl script MISA (http://pgrc.ipk-gatersleben.de/misa/) by setting the minimum number of repeats to 10, 5, 4, 3, 3, and 3 for mono-, di-, tri-, tetra-, penta-, and hexanucleotide SSRs, respectively.

### 4.4. Whole Plastid Genomes Comparison

Whole genome alignment with 10 *Primula* plastid genomes was run in MAUVE [74] under default settings to test rearrangement events across genomes.

### 4.5. Sequence Divergence Analysis

The mVISTA program [75] was used to compare the 10 *Primula* plastid genomes with *P. poissonii* as the reference. The percentages of variable characters in each coding region and non-coding region with an aligned length of more than 200 bp were calculated as described in a previous study of Poaceae species [76]. The average genetic divergences of these *Primula* plastid genomes were estimated using p-distance with MEGA6 [77]. Substitution included transition and transversion. Gaps and missing data were completely deleted.

### 4.6. Phylogenomic Analysis

To investigate the phylogenetic position of *Primula*, we used 31 complete plastid genomes (Table S3). Among them, 29 were from Ericales, and two *Hydrangea* species (*Hydrangea serrata* and *Hydrangea petiolaris*) were used as the outgroups. 76 shared protein-coding genes, codon positions 1 + 2, and codon position 3, were used to conduct the phylogenetic analysis.

Then, six datasets, including the whole plastid genomes, protein-coding regions, LSC, SSC, IRs, and introns & intergenic spacers were used to conduct the phylogenetic analysis among genus *Primula* with *A. laxa* as the outgroup.

All of the datasets were aligned with MAFFT [78] using the default settings. In order to examine the phylogenetic utility of different datasets, phylogenetic analyses were conducted using maximum likelihood (ML) and Bayesian inference (BI) methods. The ML analysis was conducted using RAxMLv7.2.8 [79] with 1000 bootstrap replicates. The GTRGAMMA model was used in all of the ML analyses, as suggested in the RAxML manual. For the BI analysis, the best substitution model was determined according to Akaike’s information criterion (AIC) with Modeltest v3.7 [80]. The BI analysis was performed using MrBayes v3.1.2 [81]. The Markov chain Monte Carlo (MCMC) algorithm was run for two million generations and the trees were sampled very 100 generations. Convergence was determined by examining the average standard deviation of the split frequencies (<0.01). The first 25% of the trees were discarded as a burn-in and the remaining trees were used to generate the consensus tree.

## Figures and Tables

**Table 1:** Plastid genomic characteristics of the 10 *Primula* species and *A. laxa.*

**Taxa**	***A. laxa*** *	***P. handeliana*** *	***P. woodwardii*** *	***P. knuthiana*** *	***P. poissonii***	***P. sinensis***	***P. veris***
Assembly reads	16,137,534	12,884,542	25,149,710	15,928,364	/	/	/
Mean coverage	293.4×	482.4×	508.3×	405.3×	/	/	/
GenBank numbers	MG181220	MG181221	MG181222	MG181223	NC_024543	NC_030609	NC_031428
Total genome size (bp)	151,942	151,081	151,666	152,502	151,664	150,859	150,856
LSC (bp)	83,078	82,785	83,325	83,446	83,444	82,064	82,048
IRs (bp)	25,970	25,200	25,290	25,604	25,199	25,535	25,524
SSC (bp)	16,924	17,896	17,761	17,848	17,822	17,725	17,760
Total GC content (%)	37.3	37	37	37	37	37.2	37.1
LSC (%)	35.2	34.9	34.9	34.9	34.9	35.2	35.1
IRs (%)	42.7	42.9	42.8	42.7	42.9	42.8	42.7
SSC (%)	30.9	30.2	30.2	30.3	30.1	30.5	30.2
Total number of genes	132	131	131	131	132	131	131
Protein-coding	87 (7)	86 (7)	86 (7)	86 (7)	86 (7)	85 (7)	86 (7)
tRNA	37 (7)	37 (7)	37 (7)	37 (7)	37 (7)	37 (7)	37 (7)
rRNA	8 (4)	8 (4)	8 (4)	8 (4)	8 (4)	8 (4)	8 (4)
Pseudogenes	/	/	/	/	infA	accD	/
**Taxa**	***P. kwangtungensis***		***P. chrysochlora***	***P. stenodonta***		***P. persimilis***	
Raw Base (G)	/		/	/		/	
Mean coverage	/		/	/		/	
GenBank numbers	NC_034371		KX668178	KX668176		KX641757	
Total genome size (bp)	153,757		151,944	150,785		152,756	
LSC (bp)	84,479		83,953	82,682		83,537	
IRs (bp)	25,855		25,460	25,182		25,753	
SSC (bp)	17,568		17,801	17,739		17,713	
Total GC content (%)	37.1		37	37.1		37.2	
LSC (%)	35		35	35		35.2	
IRs (%)	42.7		42.8	43		42.8	
SSC (%)	30.4		30.2	30.2		30.6	
Total number of genes	130		131	131		130	
Protein-coding	85 (7)		86 (7)	86 (7)		85 (7)	
tRNA	37 (7)		37 (7)	37 (7)		37 (7)	
rRNA	8 (4)		8 (4)	8 (4)		8 (4)	
Pseudogenes	/		/	/		/	

*, The four newly generated plastid genomes. LSC, large single copy region, IR, inverted repeat regions, and SSC, small single copy region.

**Table 2:** Datasets and selected model in ML and BI analysis

Datasets	Best Fit Model	Model in ML	Model in BI
76 shared protein-coding genes	TVM + I + G	GTR + G	TVM + I + G
Codon positions 1 + 2	TVM + I + G	GTR + G	TVM + I + G
Codon position 3	GTR + I + G	GTR + G	GTR + I + G
Whole plastid genomes	TVM + I + G	GTR + G	TVM + I + G
Protein-coding regions	TVM + I + G	GTR + G	TVM + I + G
Introns & intergenic spacers	TVM + I + G	GTR + G	TVM + I + G
IRs	TVM + I + G	GTR + G	TVM + I + G
LSC	GTR + I + G	GTR + G	GTR + I + G
SSC	TVM + I + G	GTR + G	TVM + I + G

## Supplementary Materials

Supplementary materials can be found at http://www.mdpi.com/1422-0067/19/4/1050/s1.
