# Supplementary material for: Comparative Plastid Genomes of Primula Species: Sequence Divergence and Phylogenetic Relationships
Source: Int J Mol Sci. 2018 Apr 1;19(4):1050. doi: 10.3390/ijms19041050 (PMC5979308; doi:10.3390/ijms19041050)
Supplement: Supplementary file 1 [file ijms-19-01050-s001.zip › supplementary materials/Supporting information.docx]

Figure S1: Genome rearrangement events in the ten *Primula* plastid genomes.

Figure S2: Phylogenetic relationship of the 31 species inferred from ML and BI analyses. (**A**) Codon positions 1 + 2. (**B**) Codon position 3. The numbers near each node are bootstrap support values and posterior probability. *Hydrangea petiolaris* and *Hydrangea serrata* were used as the outgroups.

Table S1: Sampled species used in this study.

Table S2: List of primer pairs used in sequence verification of the three *Primula* species and *A. laxa* plastid genomes.

Table S3: List of species and their accession numbers in GenBank included in the phylogenetic analysis.

Table S4: Gene composition in the *Primula* plastid genomes.

Table S5: Codon usage and relative synonymous codon usage (RSCU) value for protein-coding genes in the ten *Primula* plastid genomes.

Table S6: Base compositions for protein-coding genes in the ten *Primula* plastid genomes.

Table S7: The repeats in the ten *Primula* plastid genomes.

Table S8: Simple sequence repeats (SSRs) in the ten *Primula* plastid genomes.

Table S9: Percentages of variable characters in coding and non-coding regions.

Table S10: Genetic distance in *Primula* species.
