# Supplementary figures and images for: Comparative Plastid Genomes of Primula Species: Sequence Divergence and Phylogenetic Relationships
Source: Int J Mol Sci. 2018 Apr 1;19(4):1050. doi: 10.3390/ijms19041050 (PMC5979308; doi:10.3390/ijms19041050)

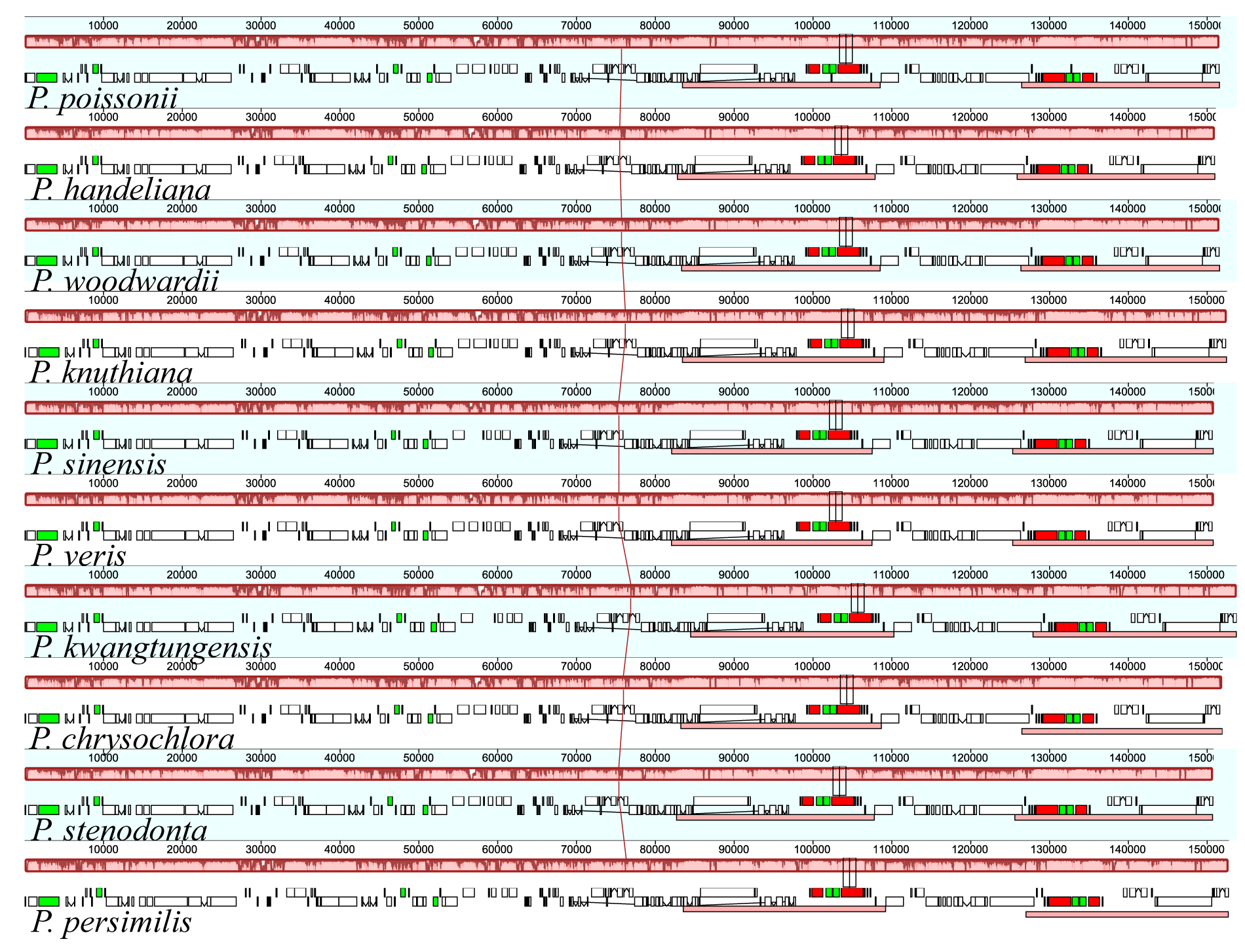

Supplement: Supplementary file 1 [file ijms-19-01050-s001.zip › supplementary materials/Figure S1.tif]

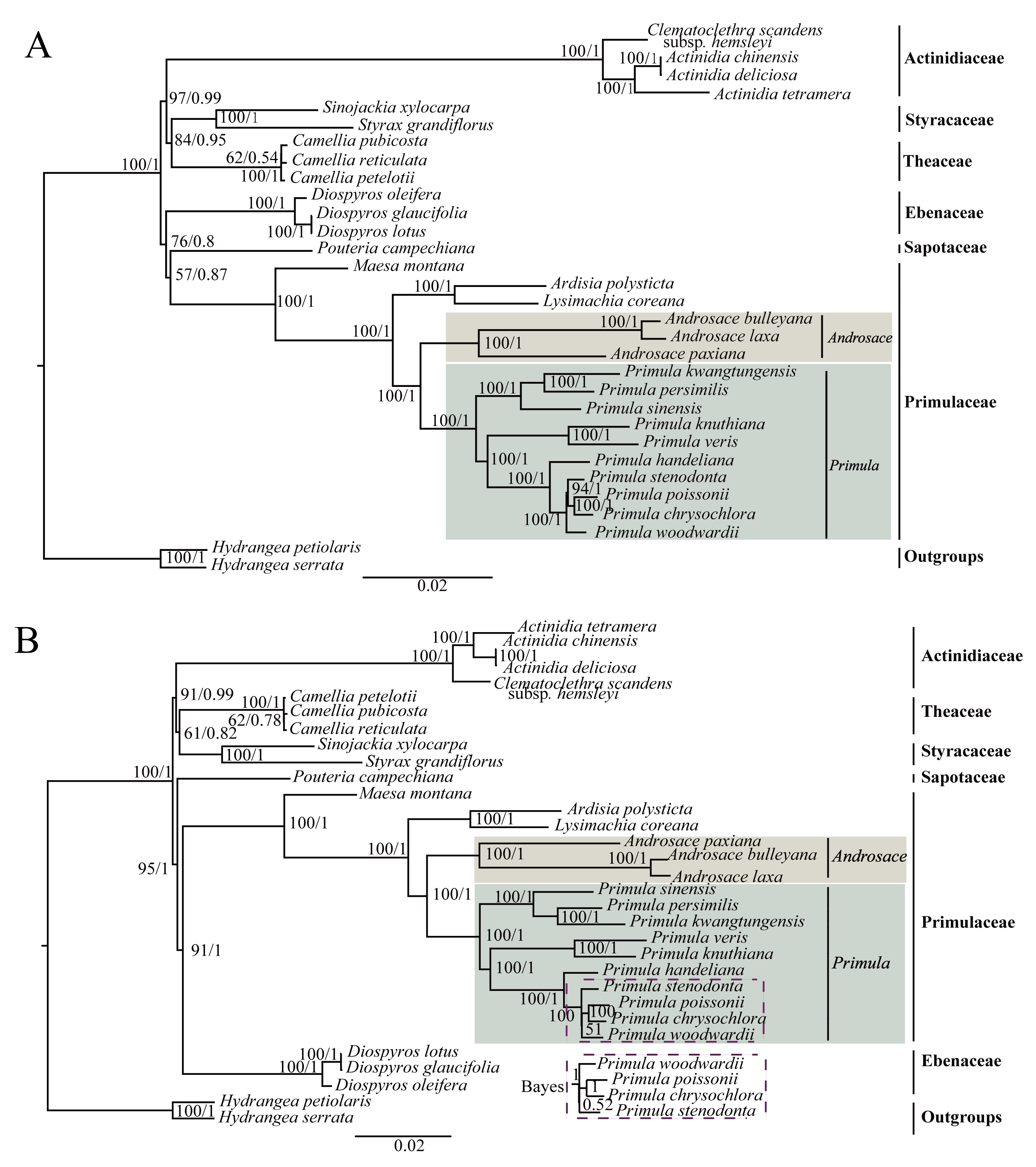

Supplement: Supplementary file 1 [file ijms-19-01050-s001.zip › supplementary materials/Figure S2.tif]
